# Ectopic Liver Tissue Attached to the Gallbladder Wall: a case report

**DOI:** 10.1186/1757-1626-2-6786

**Published:** 2009-04-29

**Authors:** Ioannis Triantafyllidis, Leonidas Papapavlou, Nikolaos Nikoloudis, Athanasios Economou, Efstathios Andreadis, Maria Chrissidou, Konstantinos Georgakis, Thomas Chrissidis

**Affiliations:** 1 Department of General Surgery, Edessa General Hospital, End of Egnatia str, 58200 Edessa, Greece; 2 Department of Pathology, Edessa General Hospital, End of Egnatia str, 58200 Edessa, Greece

## Abstract

**Introduction:**

Ectopic liver tissue is a rare entity, reported to occur in several intra-, retro- and extra- peritoneal sites, including the gallbladder. It is usually detected incidentally, during laparoscopy, laparotomy, or autopsy. Several possible mechanisms may explain the development of liver ectopia. Although ectopic liver tissue is usually asymptomatic, it behaves like orthotopic liver, developing the same pathologic conditions.

**Case presentation:**

We describe the case of a 54-year-old woman who was found to have a nodule attached to the gallbladder wall without any connection with the main liver, during an elective laparoscopic cholecystectomy for gallstone disease. The nodule was removed with the gallbladder and identified histologically as normal ectopic liver tissue.

**Conclusion:**

It would seem sensible to resect the ectopic tissue if encountered during cholecystectomy for gallstones. Laparoscopic management of ectopic liver can be feasible.

## Introduction

Ectopic liver is a rare developmental anomaly in which liver tissue is situated outside the liver and has no hepatic connection [1]. Ectopic liver tissue can occur in several different organs [2,3], but the gallbladder is the commonest site of origin. We present a case of ectopic liver attached to the gallbladder wall, encountered during an elective laparoscopic cholecystectomy, which was successfully removed with the gallbladder.

## Case presentation

A 56- year old woman with a 10 month history of multiple attacks of biliary colic was found to have gallstones on ultrasound scan and underwent a routine laparoscopic cholecystectomy.

**Figure 1:**
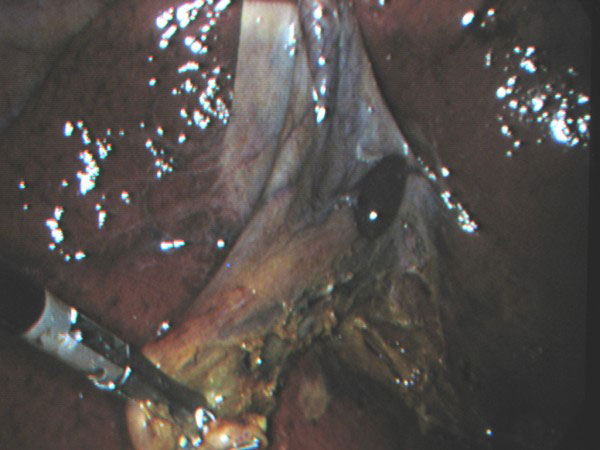
**Laparoscopic view of the gallbladder showing an encapsulated brownish mass attached to the serosal surface**.

**Figure 2:**
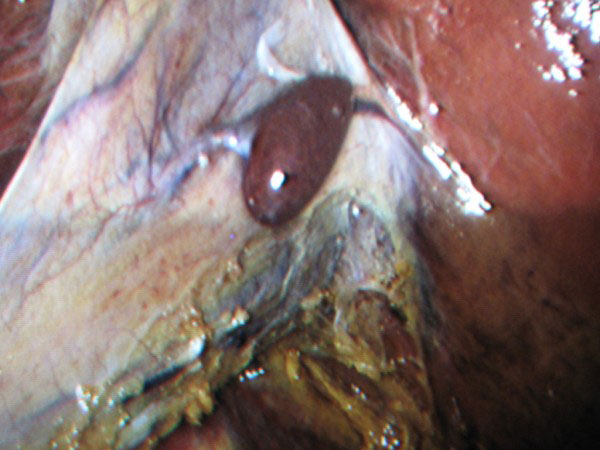
**Laparoscopic view of the gallbladder showing an encapsulated brownish mass attached to the serosal surface**.

**Figure 3:**
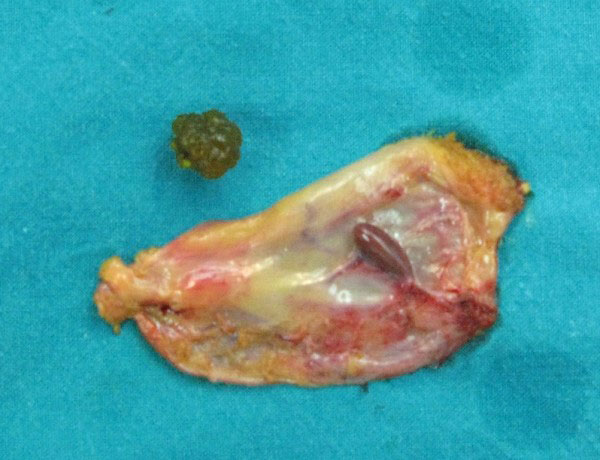
**Surgical specimen after gallbladder was opened, shows the mass (ectopic liver tissue) to protrude from gallbladder serosa and a gallstone**.

A serosal encapsulated brownish mass, attached to the serosa of the gallbladder by a fibrous pedicle (figures 1 and 2), was noted intraoperatively and was excised with the gallbladder (figure 3).

**Figure 4:**
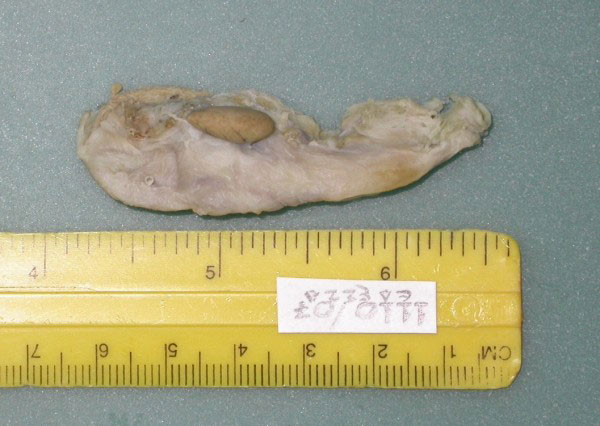
**The resected specimen fixed in formalin, shows a mass resembling normal liver, attached to the gallbladder wall**.

The mass, measuring 15 mm × 5 mm × 7 mm, looked macroscopically like normal liver tissue (figure 4).

**Figure 5:**
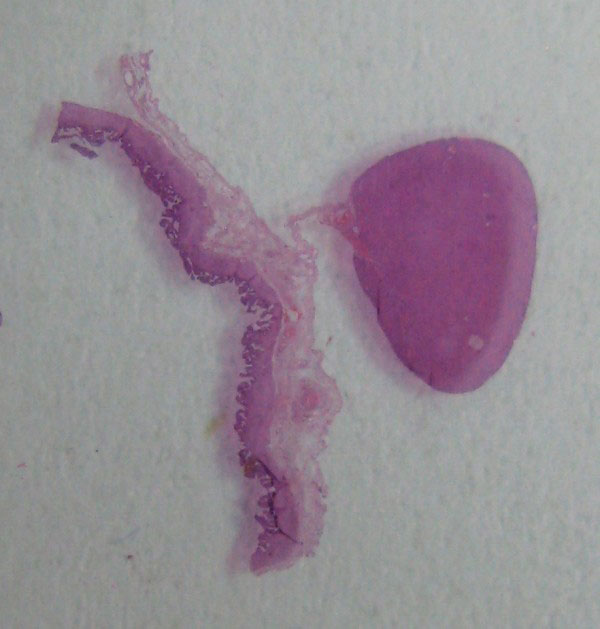
**Gross photograph of the objective plate shows the ectopic liver tissue, the gallbladder wall and the serosal stalk connecting the ectopic liver with the gallbladder**.

**Figure 6:**
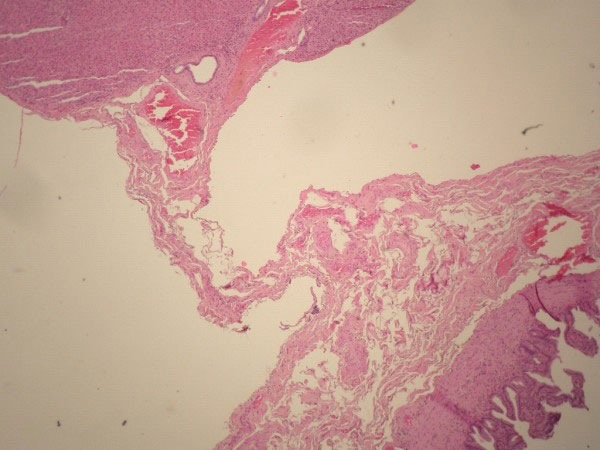
**Section of the stalk connecting the ectopic liver and the gallbladder wall**. Peritoneal mesothelial cells are lined on the stalk (hematoxylin and eosin -H&E- stain x100).

**Figure 7:**
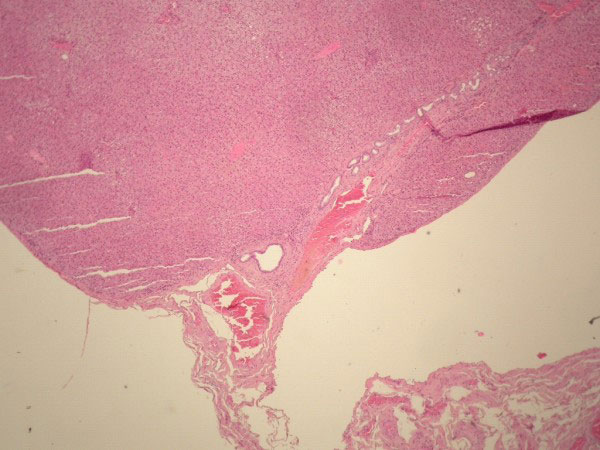
**Photomicrograph of the ectopic liver tissue (H&E x10, x100) showing liver parenchyma with normal architecture: bile ducts, arteries, veins and normal hepatocytes**. Bile ducts and vessels are seen near the serosal stalk.

**Figure 8:**
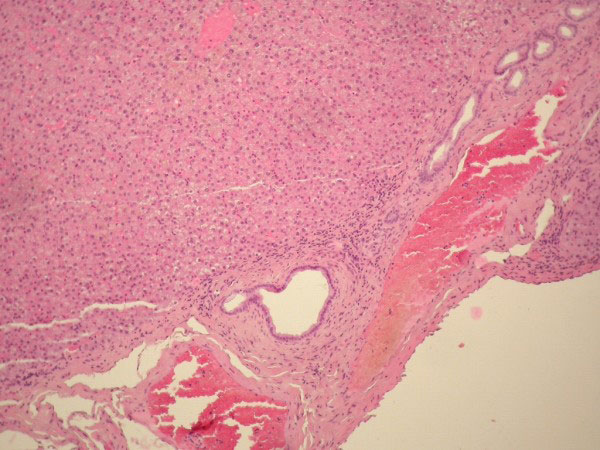
**Photomicrograph of the ectopic liver tissue (H&E x10, x100) showing liver parenchyma with normal architecture: bile ducts, arteries, veins and normal hepatocytes**. Bile ducts and vessels are seen near the serosal stalk.

**Figure 9:**
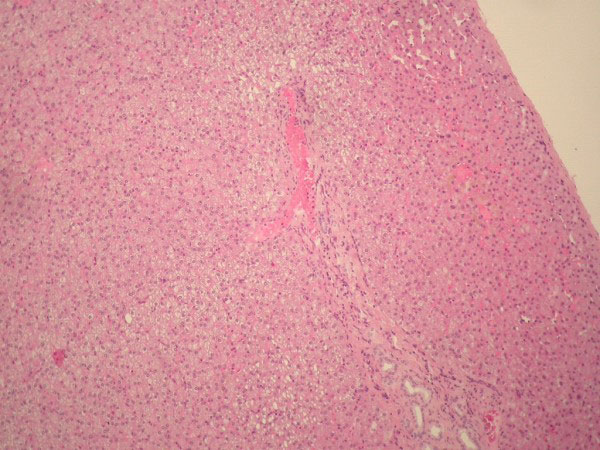
**Photomicrograph of the ectopic liver tissue (H&E x100) showing normal liver parenchyma**.

**Figure 10:**
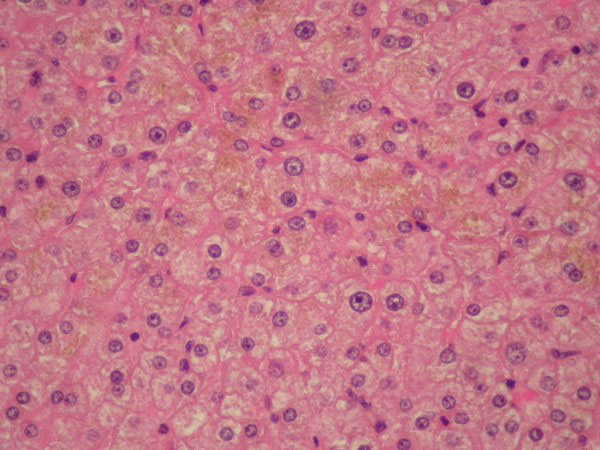
**Photomicrograph of the ectopic liver tissue (H&E x400) showing normal hepatocytes with distinctive membrane, abundant cytoplasm and regular oval nuclei**. Bile production is seen in the cytoplasm.

Histological examination revealed liver tissue containing normal tissue elements, i.e. portal tracts with bile ductules and vessels and normal hepatocytes with no disturbance in architecture or cholestasis (figures 5, 6, 7, 8, 9 and 10), suggesting normal drainage.

The patient made an uneventful postoperative recovery and was discharged home 24 hours later.

## Discussion

Anatomic anomalies of the liver have been classified as accessory lobe of the liver with attachment to native liver, and ectopic liver tissue without connection to the liver proper [1]. The term "ectopic liver" is also used, to include liver appendices attached to the native liver by a thin stalk although being fully separated from the latter [1]. Liver ectopia is the least common of the two abnormalities described. Gallbladder-associated ectopic liver is the most common location, and reports of size range from microscopic tissue to 3.7 cm [4,5].

The incidence of ectopic liver tissue attached to the gallbladder has been reported as low, but is likely to increase with the new diagnostic methods. In a 1940 review by Eiserth [6] of 5500 autopsy cases, 13 cases of ectopic liver were present, three of which were in the wall of the gallbladder. In a laparoscopic series of 1060 cases, the prevalence of ectopic liver was reported to be 0.47% [5].

Several theories have been proposed to explain the development of ectopic liver at different sites: development of an accessory lobe of the liver with atrophy or regression of the original connection to the main liver; migration or displacement of a portion of the cranial part (pars hepatica) of the liver bud to other sites; dorsal budding of hepatic tissue before the closing of the pleuroperitoneal canals; trapping of hepatocyte-destined mesenchyma in different areas and entrapment of nests of cells in the region of the foregut following closure of the diaphragm or umbilical ring [3,5,7].

The close relationship of the developing hepatic parenchymal cell cords to the pars cystica and early fetal duodenum explains why ectopic liver tissue could be found in the wall of the gallbladder, the gastrohepatic ligament, the umbilical cord, the adrenal glands, the diaphragm, the pancreas, the pylorus and the splenic capsule if a portion of the pars hepatica is displaced. Dorsal budding of hepatic tissue before closing of the pleuroperitoneal canals may explain how ectopic liver develops in the thoracic cavity, in locations such as esophagus, pericardium, intrapleural or extrapleural [3].

Although the ectopic tissue is usually attached to the serosa of the gallbladder, as in the present case, or lies within its wall, it can also occur in the gallbladder lumen [8].

Ectopic liver is sometimes associated with other congenital anomalies such as biliary atresia, agenesis of the caudate lobe, omphalocele, bile duct cyst or cardiac and conotruncal anomalies, but not when the heterotopic tissue is in the gallbladder [7,8].

Ectopic livers are supplied by an autonomous artery, not derived from the hepatic artery and some lesions do not have a portal vein system and a ductal connection with the billiary tract [9]. Ectopic liver may have its own mesentery and, depending on its location, can drain into the biliary tract, into another organ or have no drainage system [10,11]. The histological architecture of the ectopic resembles normal liver, with regular lobules, central veins, and normal portal areas in most cases [3]. In our case, ectopic liver tissue has its own mesentery and contains normal tissue elements, i.e. normal hepatocytes, arteries, veins, bile ductules with no disturbance in architecture. Drainage into the gallbladder seems likely because of the absence of bile duct dilatation or cholestasis.

The natural course of ectopic liver tissue is unpredictable. The anomaly is relatively common in the perinatal period but disappears during postnatal remodelling [12].

Symptoms occur rarely, and ectopic livers have been reported to cause recurrent abdominal pain due to torsion, compression of adjacent organs, intraperitoneal bleeding, as well as obstruction of the esophagus, portal vein, neonatal gastric outlet and pylorus [5,13]. Few cases of symptomatic ectopic liver were reported in the literature, some of which were in infants [14]. The number of reported cases of ectopic liver that gave rise to acute symptoms was even fewer.

Ectopic livers are subject to the same risk factors of disease and pathological processes that can affect the liver proper. Reported examples [5,6] include fatty infiltration of the liver, cirrhosis, chronic active hepatitis, hemosiderosis and metastatic tumor. Most importantly ectopic livers are predisposed to developing neoplastic transformation, regardless of disease or tumour in the mother liver. Small ectopic liver tissue does not have a complete functional architecture, lacking a complete vascular and ductal system and is perhaps metabolically handicapped, leading to longer exposure to various carcinogenetic factors and facilitating the carcinogenetic process [15]. Hepatocellular carcinoma develops extremely rarely in ectopic liver attached to the gallbladder [15]. A possible explanation for this difference is that ectopic liver attached to gallbladder is an anomaly occurring later during late embryogenesis and is therefore well differentiated. A consistent finding in ectopic liver is that similar changes are present in both ectopic tissue and liver proper [5].

Ectopic liver is usually an incidental finding during a laparoscopy, laparotomy or autopsy performed for unrelated reasons, most commonly for diseases of the gallbladder [5,15]. Detection of this entity before surgery or autopsy by means of imaging studies appears rare. This may be due to the small size of many ectopic livers, the failure of radiologists to be aware of this unusual entity, and the usual lack of symptoms [3]. Hepatobiliary Imino-Diacetic Acid (HIDA) scan, besides ultrasonography and computerized tomography, may be helpful in diagnosis. Colour Doppler ultrasound or angiography may demonstrate a feeding vessel. Percutaneous imaging-guided biopsy may provide a diagnosis by revealing normal liver parenchyma [3].

## Conclusions

An awareness of this entity may prevent mistaken diagnoses on those rare occasions when it is encountered. It would seem sensible to resect the ectopic tissue if encountered during cholecystectomy for gallstones, but to leave it alone if seen incidentally during other procedures. The removal of a known asymptomatic ectopic liver should be considered because of the potential for torsion and because of the increased propensity for malignancy. For obvious reasons, timely surgical treatment is necessary in the case of complicated ectopic liver. Laparoscopic management of ectopic liver can be feasible as in the case that we have presented.

## Consent

Written informed consent was obtained from the patient for publication of this case report and any accompanying images. A copy of the written consent is available for review by the Editor in Chief of this journal.

## Competing interests

The authors declare that they have no competing interests.

## Authors' contributions

IT was advising doctor, the main surgeon and involved in drafting the manuscript and revising it critically for content. LP was the pathologist involved in analyzing the specimen. NN and AE were auxiliary surgeons. EA, MC and KG were involved in revising the draft critically for content. TC, carried out strategic planning for treatment of the patient and was involved in revising the draft critically for content. All authors have given final approval of the version to be published.
